# A Mathieu function boundary spectral method for scattering by multiple variable poro-elastic plates, with applications to metamaterials and acoustics

**DOI:** 10.1098/rspa.2020.0184

**Published:** 2020-09-23

**Authors:** Matthew J. Colbrook, Anastasia V. Kisil

**Affiliations:** 1 Department of Applied Mathematics and Theoretical Physics, University of Cambridge, Wilberforce Road, Cambridge CB3 0WA, UK; 2 Department of Mathematics, The University of Manchester, Manchester, M13 9PL, UK

**Keywords:** boundary spectral methods, Mathieu functions, acoustic scattering, poro-elastic boundary conditions, metamaterials

## Abstract

Many problems in fluid mechanics and acoustics can be modelled by Helmholtz scattering off poro-elastic plates. We develop a boundary spectral method, based on collocation of local Mathieu function expansions, for Helmholtz scattering off multiple variable poro-elastic plates in two dimensions. Such boundary conditions, namely the varying physical parameters and coupled thin-plate equation, present a considerable challenge to current methods. The new method is fast, accurate and flexible, with the ability to compute expansions in thousands (and even tens of thousands) of Mathieu functions, thus making it a favourable method for the considered geometries. Comparisons are made with elastic boundary element methods, where the new method is found to be faster and more accurate. Our solution representation directly provides a sine series approximation of the far-field directivity and can be evaluated near or on the scatterers, meaning that the near field can be computed stably and efficiently. The new method also allows us to examine the effects of varying stiffness along a plate, which is poorly studied due to limitations of other available techniques. We show that a power-law decrease to zero in stiffness parameters gives rise to unexpected scattering and aeroacoustic effects similar to an acoustic black hole metamaterial.

## 1. Introduction

Motivated by many applications, there is substantial interest in solving Helmholtz scattering problems on unbounded domains with complicated boundary conditions. In this article, we consider the situation of Helmholtz scattering off (multiple) finite plates in two dimensions. When embedded in three dimensions, this corresponds to plates of infinite span but finite chord. When the geometry and boundary conditions are sufficiently simple, a successful approach for this problem is the Wiener–Hopf method [1–3]. For example, the Wiener–Hopf method allows one to capture the interaction of a semi-infinite edge with a quadrupole source and compute the far field. However, typically in such situations, one would want to model the interaction between the leading and trailing edges of a finite plate, which is important as both backscattering of the trailing-edge field by the leading edge [4] and structural resonances can be significant. There are some extensions of the Wiener–Hopf method which can deal with finite plates, but such extensions are non-generic and difficult due to the need to solve a matrix, rather than a scalar, Wiener–Hopf equation. Another common case encountered in applications, which cannot be tackled by the Wiener–Hopf method, is when physical parameters vary along the boundary of the domain. Such variations are expected to be crucial in biological applications [5] and to avoid discontinuous boundary conditions where additional scattering occurs [6]. Variation in physical parameters is also important in the study of metamaterials, such as acoustic black holes (see §5), which rely on a smooth variation of stiffness that, in the right circumstances, leads to almost 100% absorption of the incident wave energy [7,8]. Interactions of acoustic or hydrodynamic fluctuations with thin elastic structures arise in numerous other situations such as aerodynamic noise reduction [6,9–11] and the modelling of ice sheets and marine platforms in oceanography [12–15]. In all such cases, accurate and fast numerical methods are key to predicting the effect of external forces and variable parameters such as elasticity on an elastic plate, or the effect of elasticity on the radiated field, and thus crucial for providing insight into a wide range of fluid dynamical problems.

By starting with separation of variables in elliptic coordinates, we develop a boundary spectral method for scattering by multiple variable poro-elastic plates. This allows both accurate and rapid computation of the scattered field, as well as great flexibility in the boundary conditions specified on the plates. Separation of variables leads to angular Mathieu equations and radial Mathieu equations, and the solutions to these equations are the well-known Mathieu functions [16,17]. Historically, the problem of plane wave scattering of a rigid screen was first rigorously studied by Schwarzschild [18] based on the Sommerfeld half-plane problem and shortly after by Sieger [19] by employing Mathieu functions. Some numerical work based on this solution was presented in [20,21], and more recently in [22]. Extensions with different boundary conditions on elliptic shells were considered in [22,23]. Mathieu functions were also shown to be an effective tool for low-frequency scattering of a rigid (non-porous) plate in [24], where comparisons were made with semi-analytical boundary integral methods.

**Figure 1.**
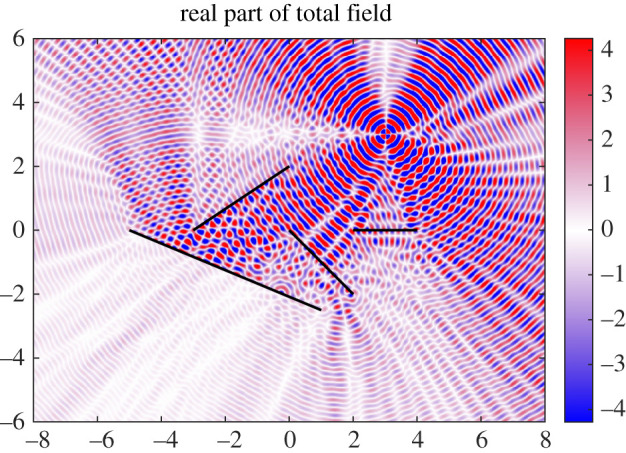
Example of scattering (real part of total field shown) with four elastic plates. The plates are emphasized for readability and we use the zero-thickness approximation in this article. The parameters correspond to (6.4), with *k*_0_ = 20 and *B* = 50. (Online version in colour.)

This article demonstrates that Mathieu functions offer a direct and rapid approach to tackle many interesting boundary value problems. To the authors’ best knowledge, the problem of acoustic scattering from multiple elastic plates with varying elasticity (or even a single plate with varying elasticity) using Mathieu functions has not been treated before. Our solution representation directly provides a sine series approximation of the far-field directivity and, unlike standard boundary methods, is easy to evaluate near the scatterers. This means that the near field can be computed efficiently and in a stable manner. These advantages mean that it is particularly good for a simple model of turbulence using Lighthill’s analogy. For example, the numerical method allows rapid and easy calculation of structural or acoustic resonances, which are generally challenging to compute when the physical parameters vary along the plate [25–27] or when sophisticated plate theories are involved [28]. To demonstrate the flexibility of the local Mathieu function expansions for arbitrarily positioned plates in two dimensions, figure 1 shows the total field for a quadrupole source scattering off four elastic plates. We also note that boundary conditions additional to those considered in §2 can easily be incorporated. It is important to point out, however, that the approach of this article cannot deal with curved boundaries which do not have a local coordinate system in which to perform separation of variables. Code for the numerical method is provided at https://github.com/MColbrook/MathieuFunctionCollocation.

Problems similar to a poro-elastic finite plate include the case of semi-infinite plates that are uniformly porous [1], or uniformly poro-elastic [11], which can be treated using scalar Wiener–Hopf techniques. These examples can be extended to more complicated porous boundary conditions [6,29], but in such cases, the analysis leads to a matrix Wiener–Hopf equation which is more difficult to solve. Elastic properties have also complexified previous numerical simulations. For example, recent work [30] (extended to three dimensions in [31]) for the scattering of a near-field source by a finite perforated elastic flat plate requires two problems to be solved; one for the structural modes of the plate which is done via a spectral method; the second for the scattering of the acoustic source which is achieved via a boundary element method (BEM). We compare our results (in the restricted case of constant porosity and constant elasticity dealt with in [30]) to those of [30] in §3c, demonstrating that separation of variables yields a faster, more robust and more accurate method for the case of a single plate. See also [32,33] for an expansion scheme of the plate deformation connected to Chebyshev polynomials that tackles the problem of a single elastic plate in a rigid baffle (our numerical scheme can handle this problem with an appropriate modification of the boundary conditions when we separate variables in §3). Another approach for these types of problems is the unified transform [34] (see also [35–38] for recent developments), a Fourier space boundary spectral collocation method which in certain cases generalizes the Wiener–Hopf method [9,39].^1^ However, using the unified transform in unbounded domains requires the setting up of several global relations by hand, which becomes complicated in complex geometries. More broadly, there has been recent interest in spectral methods to solve scattering problems that can be recast as a Riemann–Hilbert problem [43,44], though, as far as the authors are aware, such methods have not yet been applied to elastic or porous scatterers.

The structure of this article is as follows. In §2, we describe the mathematical model for a single plate. The numerical method is presented in §3, where we also compare with the boundary element method of [30]. Examples of diffraction by elastic plates of varying stiffness are presented in §4, including the peculiar effects of an acoustic black hole in §5; we are not aware of any previous studies of this effect in such plates [8]. In §6, we describe how to extend the method to multiple plates. Concluding remarks are given in §7.

## 2. Mathematical model for single plate

Suppose that an incident sound wave travels towards a plate situated at −*d* ≤ *x* ≤ *d* (where *d* > 0) and *y* = 0. The incident field will be denoted *ϕ*_I_ and the scattered field by *ϕ*. The incident pressure field is given by $p_{\text{I}}=\rho_{f}c_{0}^{2}\phi_{\text{I}}$, where *ρ*_*f*_ is the mean fluid density and *c*_0_ the speed of sound, so that throughout we deal with dimensionless fields *ϕ*_I_ and *ϕ*. We assume that *ϕ* has the usual time dependence e${\;}^{-\text{i}\omega t}$ (omitted throughout) and therefore satisfies the Helmholtz equation

$$\left(\frac{\partial }{\partial x^{2}}+\frac{\partial }{\partial y^{2}}+k_{0}^{2}\right)\phi =0,$$

where *k*_0_ = *ω*/*c*_0_ is the acoustic wavenumber for angular frequency *ω*. For instance, the pressure due to a plane wave of unit amplitude incident at angle *θ*, measured from the positive *x*-axis anticlockwise in the usual manner, corresponds to the choice

$$\phi_{\text{I}}(x,y)={\text{e}}^{-\text{i}k_{0}(x\cos\theta +y\sin\theta )}.$$

Another choice we use is a quadrupole sound source corresponding to

$$\phi_{\text{I}}(x,y)=\frac{\text{i}k_{0}^{2}}{4r_{0}^{2}}(x-x_{0})(y-y_{0})H_{2}^{\left(1\right)}(k_{0}r_{0}),$$

where (*x*_0_, *y*_0_) is the source location, $r_{0}(x,y)=\sqrt{{\left(x-x_{0}\right)}^{2}+{\left(y-y_{0}\right)}^{2}}$ is the distance to the source, and $H_{n}^{\left(1\right)}$ are Hankel functions of the first kind.

We consider poro-elastic boundary conditions. Other types of boundary conditions can also be tackled by the methods of this article (see, for example, the list of boundary conditions and physical interpretations in [45]), including non-local boundary conditions, but we stick to the following case for brevity. For completeness, we have also provided an electronic supplementary material detailing the implementation for rigid porous plates.

We consider a poro-elastic plate with evenly-spaced circular apertures of radius *R*, Rayleigh conductivity of *K*_*R*_ = 2*R*, and fractional open area *α*_*H*_ = *NπR*^2^ (where *N* is the number of apertures per unit area) [46]. The plate deformation is given by $\eta (x){\text{e}}^{-\text{i}\omega t}$ (the time dependence is again assumed and omitted) and *η*(*x*) satisfies the thin-plate equation

2.1 $$B_{0}(x)\eta (x)+\sum_{l=1}^{4}B_{l}(x)\frac{\partial^{l}\eta }{\partial x^{l}}(x)=-\rho_{f}c_{0}^{2}\left(1+\frac{4\alpha_{H}}{\pi }\right)[\phi ](x).$$

We use the notation *ϕ*(*x*, 0 + ) and *ϕ*(*x*, 0 − ) to denote the values of the field just above and just below the plate, respectively. For notational convenience, the jump *ϕ*(*x*, 0 + ) − *ϕ*(*x*, 0 − ) in *ϕ* across the plate is denoted by [*ϕ*](*x*). We have written (2.1) in general form since the collocation method can deal with such general boundary conditions. In later sections, we consider specific models of flexural waves along a thin plate of varying thickness. For details of this model derivation see [11]. There is also a kinematic condition on the plate

2.2 $$\frac{\partial \phi }{\partial y}{|}_{y=0}+\frac{\partial \phi_{\text{I}}}{\partial y}{|}_{y=0}=k_{0}^{2}[(1-\alpha_{H})\eta +\alpha_{H}\eta_{a}],$$

where $\eta_{a}=K_{R}[\phi ]/(\pi k_{0}^{2}R^{2})$ is the average fluid displacement in the apertures. Finally, there are two more boundary conditions at each end of the plate. For each end, say at *x* = *x*_0_, of the elastic plate, the edge is either

$$\text{free }:\;\eta^{\prime \prime }(x_{0})=\eta^{\prime \prime \prime }(x_{0})=0,\;\text{or clamped}:\;\eta (x_{0})=\eta^{\prime }(x_{0})=0.$$

Note that when |*x*| > *d*, *ϕ*(*x*, 0) = 0. The solution *ϕ* is also required to satisfy the Sommerfeld radiation condition for outgoing waves at infinity given by

$$\lim_{r\to \infty }r^{-1/2}\left(\frac{\partial \phi }{\partial r}-\text{i}k_{0}\phi \right)=0,\;\text{where }r=\sqrt{x^{2}+y^{2}}.$$

## 3. Single plate solution

### (a) Expansion of solution in Mathieu functions

**Figure 2.**
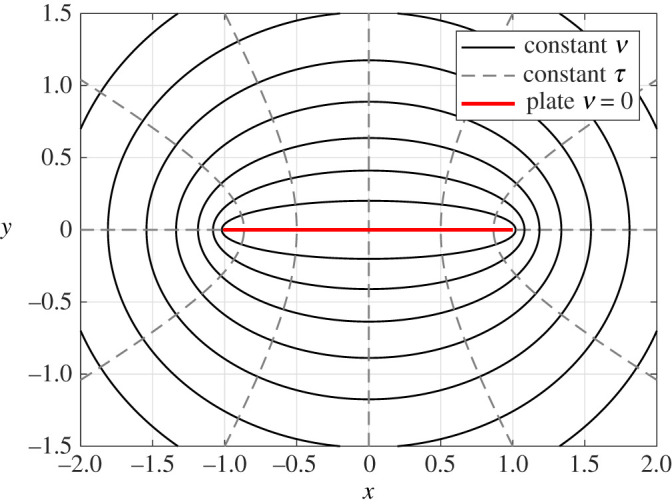
Elliptic and Cartesian coordinates for *d* = 1. (Online version in colour.)

The solution *ϕ* is an odd function in the variable *y* and hence we can consider solving the PDE system in the upper-half plane {(*x*, *y*):*y* > 0}. First, we introduce elliptic coordinates via *x* = *d*cosh (*ν*)cos (*τ*), *y* = *d*sinh (*ν*)sin (*τ*), where, with an abuse of notation, we write functions of (*x*, *y*) also as functions of (*ν*, *τ*). Elliptic coordinates for *d* = 1 are displayed in figure 2. The appropriate domain then becomes *ν* ≥ 0 and *τ* ∈ [0, *π*]. To simplify the formulae, we let $Q=d^{2}k_{0}^{2}/4$. Separation of variables leads to the expansion

3.1 $$\phi (\nu ,\tau )=\sum_{m=1}^{\infty }a_{m}{\text{se}}_{m}(\tau ){\text{Hse}}_{m}(\nu ),$$

where se_*m*_(*τ*) = se_*m*_(*Q*;*τ*) denote sine-elliptic functions and Hse_*m*_(*Q*;*ν*) = Hse_*m*_(*ν*) denote Mathieu–Hankel functions. A full derivation is provided in the electronic supplementary material.

The functions se_*m*_ are expanded in a sine series as

3.2 $${\text{se}}_{m}(\tau )=\sum_{l=1}^{\infty }B_{l}^{\left(m\right)}\sin(l\tau ).$$

This Fourier series converges absolutely and uniformly on all compact sets of the complex plane [17] and we find the coefficients $B_{l}^{\left(m\right)}$ via a simple Galerkin method. The convergence to the eigenvalues and eigenfunctions depends on the parameter *Q*, in general being slower for larger *Q*. However, the convergence is exponential, yielding machine precision for small truncation parameter *n*, even for very large *Q* [42].

The functions Hse_*m*_(*ν*) can be expanded using Bessel functions [16,17]:

3.3 $$\begin{array}{rl} {\text{Hse}}_{m}(\nu ) & =\sum_{l=1}^{\infty }\frac{{\left(-1\right)}^{l+m}B_{l}^{\left(m\right)}}{C_{m}}\\ & \;\times \left[J_{l-1}({\text{e}}^{-\nu }\sqrt{Q})H_{l+p_{m}}^{\left(1\right)}({\text{e}}^{\nu }\sqrt{Q})-J_{l+p_{m}}({\text{e}}^{-\nu }\sqrt{Q})H_{l-1}^{\left(1\right)}({\text{e}}^{\nu }\sqrt{Q})\right], \end{array}$$

where *p*_*m*_ = 1 if *m* is even and *p*_*m*_ = 0 if *m* is odd. Here *J*_*n*_ denotes the Bessel function of the first kind of order *n* and we remind the reader that $H_{n}^{\left(1\right)}$ denotes the Hankel function of the first kind of order *n*. The series in (3.3) converges absolutely and uniformly on all compact sets of the complex plane [17]. We choose the normalization constants *C*_*m*_ such that Hse_*m*_′(0) = 1. The terms in the series (3.3) can easily be evaluated for small *l*. However, for large *l*, the terms in the series suffer from underflow and overflow associated with cancellations between the Bessel and Hankel functions. For large *l* and fixed $x\in R_{>0}$, we use the asymptotics

$$J_{l}(x)=\sum_{j=0}^{q}\frac{{\left(-1\right)}^{j}}{j!(j+l)!}{\left(\frac{x}{2}\right)}^{2j+l}+O\left(\frac{1}{(q+l+1)!}\right)$$

and

$$H_{l}^{\left(1\right)}(x)=\frac{-\text{i}}{\pi }{\left(\frac{2}{x}\right)}^{l}\sum_{j=0}^{q}\frac{(l-j-1)!}{j!}{\left(\frac{x}{2}\right)}^{2j}+O((l-(q+2))!),$$

valid as *l* → ∞. For fixed a,b∈Z, this gives the asymptotic form

$$\begin{array}{rl} & J_{l+a}({\text{e}}^{-\nu }\sqrt{Q})H_{l+b}^{\left(1\right)}({\text{e}}^{\nu }\sqrt{Q})\\ & =\frac{-\text{i}}{\pi }{\left(\frac{\sqrt{Q}}{2}\right)}^{a-b}{\text{e}}^{-\nu (2l+a+b)}\left[\sum_{j=0}^{q}\frac{{\left(-1\right)}^{j}(l+a)!}{j!(j+l+a)!}{\left(\frac{{\text{e}}^{-\nu }\sqrt{Q}}{2}\right)}^{2j}\right]\\ & \;\;\;\;\;\;\times \left[\sum_{j=0}^{q}\frac{(l+b-j-1)!}{j!(l+a)!}{\left(\frac{{\text{e}}^{\nu }\sqrt{Q}}{2}\right)}^{2j}\right]+O(l^{-(q+2)}) \end{array}$$

**Figure 3.**
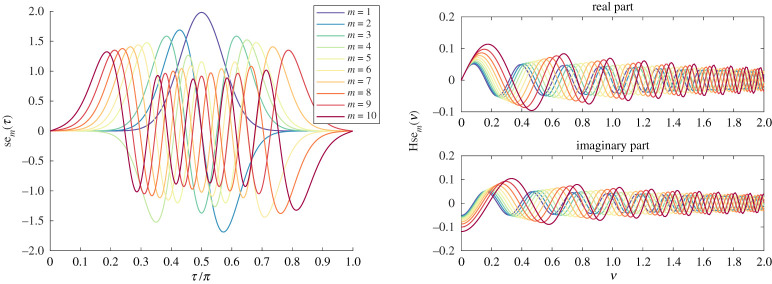
First 10 Mathieu functions used for separation of variables for *k*_0_ = 20. (Online version in colour.)

We found this to be an excellent approximation for large *l*. It can also be accurately evaluated for moderate *q* since the terms (*l* + *a*)!/(*j* + *l* + *a*)! and (*l* + *b* − *j* − 1)!/(*l* + *a*)! can be evaluated as products of *j* and |*j* + 1 + *a* − *b*| terms, respectively. In what follows, we typically used this asymptotic form when *l* > 100 and took up to *q* = 5 terms. When plotting errors of our method, we were careful to compare against converged computations for which the series (3.3) was evaluated directly using extended precision (such checks were the only place where we made use of extended precision). Figure 3 shows the first 10 eigenfunctions and Mathieu–Hankel functions for *k*_0_ = 20 computed to machine precision.

We use the boundary conditions to solve for the unknown coefficients *a*_*m*_, after which the solution can be evaluated anywhere in the (*x*, *y*) plane. Of particular interest is the far-field directivity, *D*(*θ*), which is defined via

3.4 $$\phi (r,\theta )∼D(\theta )\frac{{\text{e}}^{\text{i}wr}}{\sqrt{r}},\;\text{as }\;r\to \infty ,$$

where (*r*, *θ*) are the usual polar coordinates. Given the Bessel function expansion of Hse_*m*_(*ν*) in (3.3), we can directly compute *D*(*θ*) from (3.1) using asymptotics of Bessel functions (for large arguments, not large order as was used previously above). In the appropriate limit, *τ* becomes the polar angle *θ*, whereas *ν* becomes cosh ^−1^(*r*/*d*) (in the far field the confocal ellipses can be approximated by concentric circles, see figure 2). We therefore have

3.5 $${\text{Hse}}_{m}(\nu )∼\frac{{\left(-1\right)}^{m+1}}{C_{m}\sqrt{\pi r\sqrt{Q/d^{2}}}}\exp\left(\left[2r\sqrt{Q/d^{2}}-\frac{(p_{m}+1)\pi }{2}-\frac{\pi }{4}\right]\text{i}\right)B_{1}^{\left(m\right)},\;\text{as }r\to \infty ,$$

and hence

3.6 $$D(\theta )=\sqrt{\frac{2}{\pi k_{0}}}\sum_{m=1}^{\infty }\frac{a_{m}B_{1}^{\left(m\right)}}{C_{m}}\exp\left(\frac{(2p_{m}-3)\pi }{4}\text{i}\right){\text{se}}_{m}(\theta ).$$

An advantage of our approach is that, for the case of a single plate, we implicitly compute a sine series for the far-field directivity *D*(*θ*) through the sine-elliptic functions se_*m*_(*θ*) given by (3.2).

### (b) Employing the boundary conditions

We adopt a spectral collocation method for finding the unknown coefficients in the expansion (3.1). Throughout, we denote the approximate coefficients by ${\tilde{a}}_{m}$. When numerically solving the resulting linear system, we found it helpful to precondition by rescaling to ensure that each row of the resulting matrix has a constant *l*^1^ vector norm.

We truncate the expansion (3.1) to *M* terms and supplement the expansion of *ϕ* with an expansion of the plate deformation *η* in terms of Chebyshev polynomials of the first kind

$$\eta (x)=\sum_{j=0}^{\infty }b_{j}T_{j}\left(\frac{x}{d}\right).$$

We truncate this expansion to *N* terms for approximate coefficients ${\tilde{b}}_{j}$. The relation (2.1) becomes

3.7 $$\begin{array}{rl} & \sum_{j=0}^{N-1}{\tilde{b}}_{j}\sum_{l=0}^{4}\frac{B_{l}(x)}{d^{l}}T_{j}^{\left(l\right)}\left(\frac{x}{d}\right)\\ & \;+2\rho_{f}c_{0}^{2}\left(1+\frac{4\alpha_{H}(x)}{\pi }\right)\sum_{m=1}^{M}{\tilde{a}}_{m}{\text{se}}_{m}\left(\cos^{-1}\left(\frac{x}{d}\right)\right){\text{Hse}}_{m}(0)=0. \end{array}$$

The kinematic relation (2.2) becomes

3.8 $$\begin{array}{rl} & \sqrt{d^{2}-x^{2}}\cdot \frac{\partial \phi_{\text{I}}}{\partial y}(x)+\sum_{m=1}^{M}{\tilde{a}}_{m}{\text{se}}_{m}\left(\cos^{-1}\left(\frac{x}{d}\right)\right)\left[1-\frac{4\alpha_{H}(x){\text{Hse}}_{m}(0)}{\pi R(x)}\sqrt{d^{2}-x^{2}}\right]\\ & \;=k_{0}^{2}(1-\alpha_{H}(x))\sqrt{d^{2}-x^{2}}\sum_{j=0}^{N-1}{\tilde{b}}_{j}T_{j}\left(\frac{x}{d}\right). \end{array}$$

We collocate the kinematic condition (3.8) at the points

$$\left\{d\cos\left(\frac{2j-1}{2M}\pi \right):j=1,\ldots ,M\right\},$$

which correspond to (rescaled) Chebyshev points in Cartesian coordinates and equally spaced points in elliptic coordinates [47,48]. However, for (3.7), we choose *N* − 4 Chebyshev points and supplement this system with four relations enforcing the boundary conditions at ±*d*. This gives rise to a coupled square (*M* + *N*) × (*M* + *N*) linear system for the unknown coefficients $\left\{{\tilde{a}}_{m},{\tilde{b}}_{j}:m=1,\ldots ,M,j=0,\ldots ,N-1\right\}$.

### (c) Comparison with elastic boundary element method

In this section, we analyse the numerical performance of the proposed method with *constant* physical parameters. Further examples where parameters vary will be given in later examples. A comparison between our method and the unified transform for a rigid porous plate can be found in [42].

We compare the proposed collocation method with the BEM of [30], which deals with constant porosity and elasticity. The method of [30] first computes the spectral modes of the fourth-order derivative operator (acting on the left-hand side of (2.1)), before recasting the boundary conditions in terms of these vibration modes of the plate, and then solving the resulting boundary element scheme. In this section, we shall be consistent with the set-up of [30] and consider a plate that lies along {(*x*, 0):*x* ∈ [0, 1]}, is clamped at *x* = 0, and free to move at *x* = 1. To compare with the parameters of [30], for a plate of mass *m* per unit area and effective plate stiffness $\bar{B}$, we define^2^ the coincidence frequency

$$\omega_{c}={\left(\frac{(1-\alpha_{H})mc_{0}^{4}}{\bar{B}}\right)}^{1/2},$$

the vacuum bending wave Mach number

$$\Omega ={\left(\frac{\omega }{\omega_{c}}\right)}^{1/2}=\frac{k_{0}}{k_{B}},$$

and the intrinsic fluid-loading parameter

$$\epsilon =\frac{\rho_{f}k_{0}}{(1-\alpha_{H})mk_{B}^{2}}.$$

Note that since we are considering constant parameters in this subsection, *ω*_*c*_, Ω and *ϵ* are constant. After a suitable rescaling with the plate length (and, with an abuse of notation, keeping the same notation for physical parameters), the non-dimensionalized boundary conditions become

3.9 $$\left(1-\alpha_{H})\frac{\partial^{4}\eta }{\partial x^{4}}-\frac{k_{0}^{4}}{\Omega^{4}}\eta =-\left(1+\frac{4\alpha_{H}}{\pi }\right)\frac{\epsilon }{\Omega^{6}}k_{0}^{3}[\phi \right]$$

and

3.10 $$\frac{\partial \phi }{\partial y}{|}_{y=0}+\frac{\partial \phi_{\text{I}}}{\partial y}{|}_{y=0}=(1-\alpha_{H})k_{0}^{2}\eta +\frac{2\alpha_{H}}{\pi R}[\phi ].$$

A broad parametric study of how our collocation approach compares to BEM would be an exhaustive task. Instead, we provide some comparisons pertinent to the general performance of both methods. We therefore set *R* = 10^−3^, *α*_*H*_ = 2 × 10^−3^ and *ϵ* = 0.0021 throughout this section (representative of an aluminium plate in air [46]). We compare both methods for computing the far-field directivity (computed by measuring the scattered field at radius 100 for BEM), using a discrete relative *L*^2^ error defined by

$$\sqrt{\frac{\sum_{i}|\tilde{D}(\theta_{i})-D(\theta_{i}){|}^{2}}{\sum_{i}|D(\theta_{i}){|}^{2}}}.$$

Here, $\tilde{D}$ is the computed directivity and *D* the true directivity which is estimated via a converged computation with larger *M* and *N* for our method, and a larger number of boundary elements and modes for BEM. The *θ*_*i*_ are taken to be 201 equally spaced points covering the interval [0, *π*]. Consistent with [30], we consider the case of placing a quadrupole at (*x*, *y*) = (1, 0.01) and compute the resulting far field of *ϕ*. We chose to compare the accuracy of computing the far-field directivity as opposed to the jump in pressure across the plate since the numerical approach of [30] adopts a small but positive plate thickness (however, we also obtain similar qualitative results for other physical quantities of interest). Therefore, we do not expect exact agreement between the BEM and our collocation approach (which deals with plates of zero thickness).

**Figure 4.**
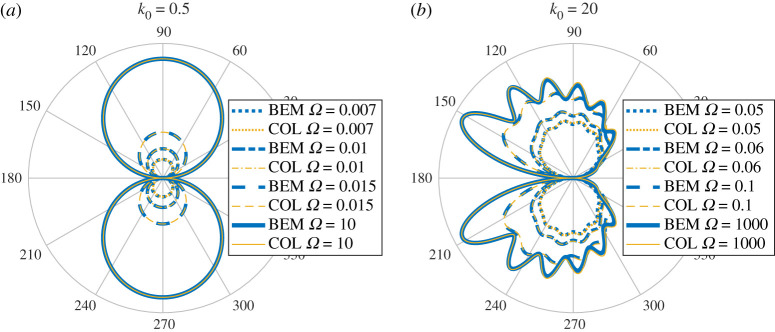
(*a*) Comparison of |*D*(*θ*)| for elastic BEM (BEM) and Mathieu function collocation (COL) for *k*_0_ = 0.5. (*b*) Same but for *k*_0_ = 20. (Online version in colour.)

**Figure 5.**
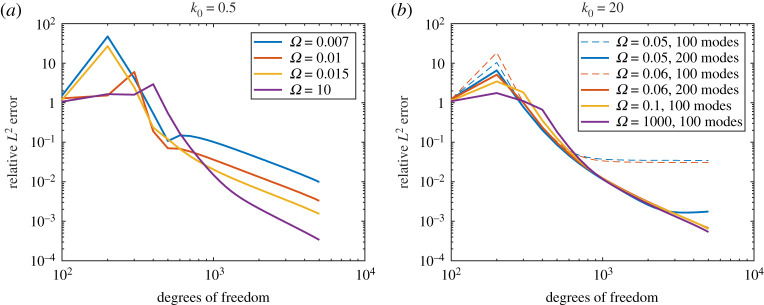
(*a*) Convergence of elastic BEM for *k*_0_ = 0.5 (100 modes). (*b*) Same but for *k*_0_ = 20 (number of modes shown). (Online version in colour.)

**Figure 6.**
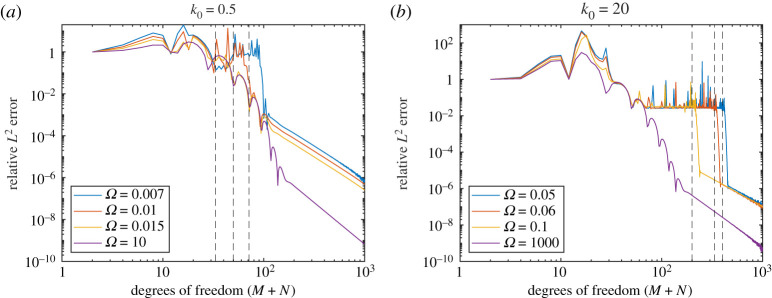
(*a*) Convergence of Mathieu function collocation for *k*_0_ = 0.5. The vertical dashed lines are positioned at the bending wavenumbers *k*_*B*_ = *k*_0_/Ω (which is too small to plot for Ω = 10). (*b*) Same but for *k*_0_ = 20. (Online version in colour.)

Figure 4 shows |*D*(*θ*)| for various Ω and *k*_0_. These show excellent agreement between both methods (we used *M* = *N* for the Mathieu function collocation method). There is a slight deviation for *k*_0_ = 20 and Ω = 0.05 due to the non-zero plate thickness in BEM (this is expected to make more of a difference for larger *k*_0_ and smaller Ω). Figure 5 shows the convergence of BEM (default 100 modes) as a function of the number of degrees of freedom of the linear system. We see quite slow algebraic convergence (typical of standard BEM). For small *k*_0_, the errors are smaller for larger Ω as the plate becomes more rigid. This was less pronounced for larger *k*_0_. However, in this case, for smaller Ω we needed a larger number of modes for the error not to plateau. This is expected since, as a rough heuristic, the number of modes needed scales as the bending wavenumber *k*_*B*_ = *k*_0_/Ω. Figure 6 shows the convergence of our Mathieu function collocation method, where we have also plotted the bending wavenumbers. For each set of parameters, there is a region of algebraic convergence (roughly cubic) once the number of degrees of freedom is of the order *k*_*B*_. There is also an initial region of rapid convergence (typical of spectral methods) most pronounced for larger Ω. The Mathieu function approach achieves errors several orders of magnitude smaller than BEM and for much fewer degrees of freedom.

**Figure 7.**
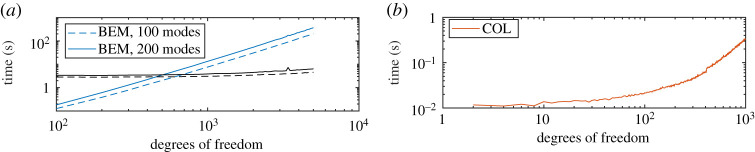
(*a*) Times taken for elastic BEM to set up and solve the linear system in blue, the precomputation of the vibrational modes are shown as the black lines. (*b*) Same but for Mathieu function collocation, where we have now included the time taken to compute the coefficients in the expansion (3.2). Note the difference in orders of magnitude on the horizontal and vertical axes—the Mathieu function collocation approach is much faster. The slight jump around *N* = 200 (400 d.f.) for the Mathieu function method is due to the introduction of the asymptotic series to compute Bessel functions of large order (the largest order scales as *N*/2 due to even and odd splitting). (Online version in colour.)

Finally, figure 7 shows the average times of the methods implemented on a 5-year-old laptop, including to evaluate the far field. The Mathieu function approach is much faster (see the different scales on the vertical and horizontal axes), even when the size of the linear systems are the same. A possible reason for this is the implementation of the BEM code, however, as demonstrated in figures 5 and 6, much smaller system sizes are needed for a given accuracy when using the collocation method. For BEM, we have shown separately the times taken to compute the vibrational modes and also to set up and solve the linear system. When using BEM, the vibrational modes do not need to be recomputed for different parameters (assuming enough modes are included to capture the oscillations). However, the precomputation of the coefficients in the expansion (3.2) via a symmetric tridiagonal eigenvalue, which needs to be performed for each value of *k*_0_ in the Mathieu function approach, takes negligible time compared to solving the linear system for large *M*.

Though we do not repeat the results here, a relative accuracy of approximately three digits and runtime of a few seconds (including evaluation) was reported in [9] for similar parameters using the unified transform. Therefore, our approach in this article is also faster and more accurate than the unified transform implemented in [9,49].

## 4. Diffraction by an elastic plate of varying thickness

For the rest of this article, we consider the choices

4.1 $$\begin{array}{rl} B_{4}(x) & =(1-\alpha_{H})\bar{B}(x),\;B_{3}(x)=2(1-\alpha_{H})\frac{\text{d}}{\text{d}x}\bar{B}(x),\\ B_{2}(x) & =(1-\alpha_{H})\frac{{\text{d}}^{2}}{\text{d}x^{2}}\bar{B}(x),\;B_{1}=0,\;B_{0}(x)=-(1-\alpha_{H})m(x)\omega^{2}, \end{array}$$

where the effective plate stiffness is $\bar{B}=[1-2\alpha_{H}\nu /(1-\nu )]B$, the plate has mass *m*(*x*) per unit area and bending stiffness *B*, and *ν* denotes the Poisson ratio of the plate material. This models flexural waves on a thin plate [46] and we also allow the bending stiffness, *B*(*x*), to vary across the plate. Namely, for a plate of varying thickness *h*(*x*) such that the wavelength of the flexural motion is much larger than *h*, the bending stiffness is given by [46,50]

4.2 $$B(x)=\frac{Eh{\left(x\right)}^{3}}{12(1-\nu^{2})},$$

where *E* is Young’s modulus. We take *ν* = 0.35 and *E* = 69 × 10^9^ Pa, typical of an aluminium plate. We also take *m*(*x*) = *m*_0_ *h*(*x*) where *m*_0_ is such that the average of *m* over the plate is 1 (taking typical values for aluminium in air from [30]), *c*_0_ = 343 ms^−1^ (speed of sound in air) and *ρ*_*f*_ = 1.23 kgm^−3^ (standard air density). Unless otherwise stated, *R* = 0.01, and *α*_*H*_ = 0.03 for a plate with *d* = 1 (lying between −1 and 1).

Here, we investigate how different variations in the plate thickness *h*(*x*) influence the scattered field. We define a functional *P*, proportional to the total above-plate scattered sound power

4.3 $$P=\int_{0}^{\pi }|D(\theta ){|}^{2}\;\text{d}\theta ,$$

where *D*(*θ*) is defined in (3.4). For the experiments in §4a and §4b, we were able to compute *P* with relative error bounded by 10^−10^ over a broad range of frequencies. We tested convergence against larger *M* = *N*, particularly for larger *k*_0_ where more terms in the expansion are needed. One potential application of the new numerical method is that it allows rapid and easy calculation of resonances, which are generally challenging to compute when the physical parameters vary along the plate [25–27] or when sophisticated plate theories are involved [28].

### (a) Linear variation

Consider first a linear variation in the plate thickness for a plate clamped at both endpoints with

**Figure 8.**
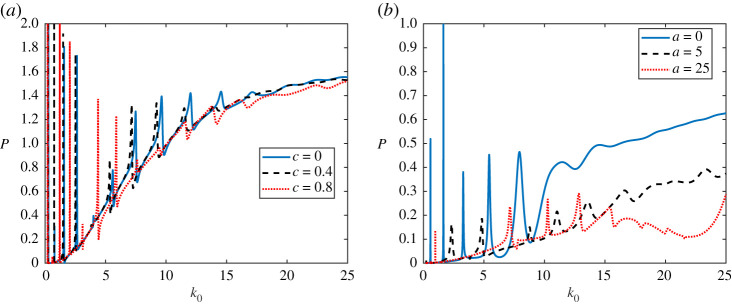
Results for linear and periodic variations. (*a*) Far-field power *P* for different acoustic wavenumer *k*_0_ and for an incoming plane wave of incidence angle *π*/3. The three different lines correspond to different values of *c* in the function *h*(*x*) in (4.4). (*b*) Far-field power *P* for different acoustic wavenumer *k*_0_ and for an incoming plane wave of incidence angle *π*/8. The three different lines correspond to different values of *a* in the function *h*(*x*) in (4.5). (Online version in colour.)

4.4 h(x)=0.004(1−cx),

for different *c*. The sound power *P* is shown in figure 8*a* for an incident plane wave of angle *π*/3. Looking at the constant stiffness (*c* = 0), the most apparent feature is the presence of resonance peaks. These resonant contributions have decreasing power and increasing frequency width as the frequency increases [51]. The resonance peaks of a fluid-loaded plate are known to be slightly lower than the *in vacuo* plate resonance wavenumbers [30]. We have found that varying *c* does not significantly change the sound power *P* variation for different acoustic wavenumbers. The only observed effect is the shift in the resonance peaks, which is small for small *c* and larger for larger *c* (as expected, smaller modifications of *h* lead to smaller changes in *P*). Hence it is found that, similarly to the introduction of fluid-loading, linear variation in *h* (and monotonic changes in *B*) changes the position of the resonance peaks in figure 8.

### (b) Periodic variation

Next, we consider the case where the thickness varies periodically for a plate clamped at both endpoints with

4.5 h(x)=0.006(1.1−sin⁡(ax)),

where the constant *a* varies. The sound power is shown in the right of figure 8 for an incident plane wave of angle *π*/8. The periodic structure of the plate significantly alters the shape of *P* as a function of *k*_0_. A reduction or increase in *P* for a specific frequency range is possible but is sensitive to the angle of the incident wave. Hence this is not studied here. The shape of the resonance response is observed to change consistently and to be angle independent. Experimenting with different *a* and angle of incidence, we found that, for large *a* and for 5 < *k*_0_ < 15, the effect of resonance is decreased and acoustic smoothing seems to occur. For frequencies between 15 < *k*_0_ < 25 increasing *a* also decreases *P*. Hence, periodically structuring the plate thickness has the potential to be used to control resonances and also decrease radiated power.

## 5. Acoustic black hole

We next consider the case of an acoustic black hole. These are new physical objects, introduced and investigated over the last 15 years or so [7,8,52–56], that under certain circumstances can absorb almost 100% of the incident wave energy. Acoustic black holes have been investigated mainly for flexural waves in thin plates, where the local thickness varies according to a power law, with the power-law exponent being greater than or equal to 2. Here, we explore their properties in acoustic scattering. Whereas previous work considers incident waves that originate inside the plate/wedge, we consider the interaction of such a plate with an incident field.

### (a) Incident plane wave

In this example, we take *α*_*H*_ ≡ 0 (i.e. zero porosity), and take the plate to be clamped at both endpoints. The thickness is chosen to vary according to

**Figure 9.**
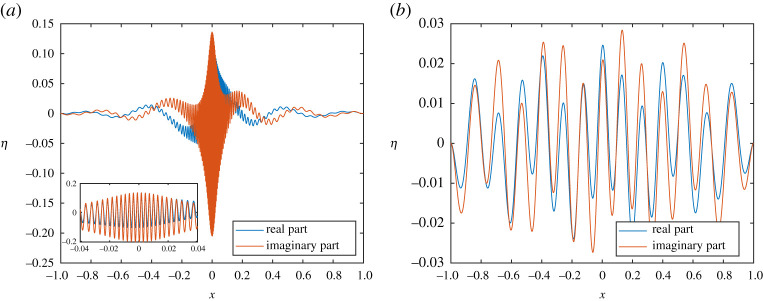
(*a*) Plate displacement, *k*_0_ = 20, for an incoming plane wave of angle 3*π*/4 for acoustic black hole with *h*_0_ = 10^−6^. The magnified section shows the oscillatory waves near *x* = 0. (*b*) Results for *h*_0_ = 10^−3^. (Online version in colour.)

**Figure 10.**
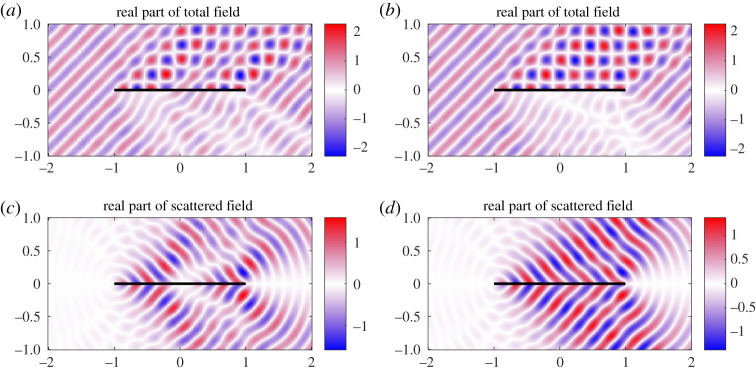
(*a*,*c*) Near field, *k*_0_ = 20, for an incoming plane wave of angle 3*π*/4 for acoustic black hole with *h*_0_ = 10^−6^. (*b*,*d*) Results for *h*_0_ = 10^−3^. (Online version in colour.)

**Figure 11.**
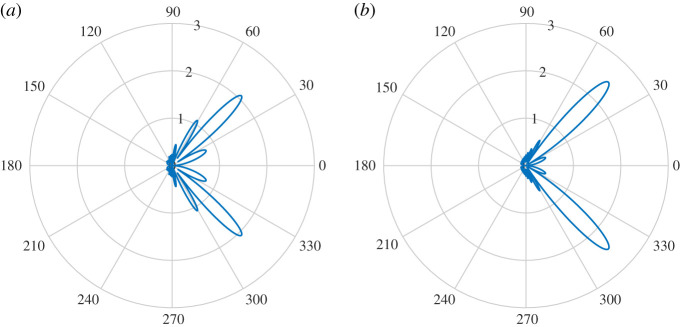
(*a*) Magnitude of the far-field directivity |*D*(*θ*)|, *k*_0_ = 20, for an incoming plane wave of angle 3*π*/4 for acoustic black hole with *h*_0_ = 10^−6^. (*b*) Results for *h*_0_ = 10^−3^. (Online version in colour.)

**Figure 12.**
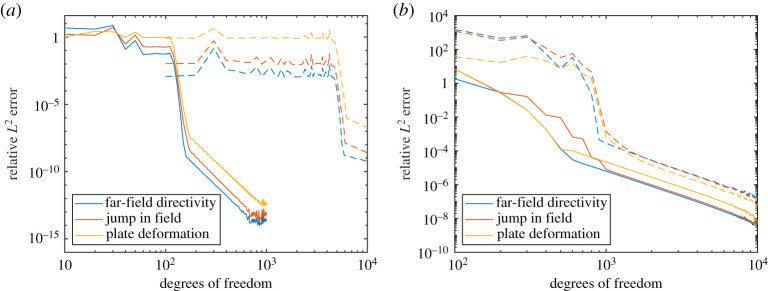
(*a*) Convergence for incident plane wave. The dotted lines are for *h*_0_ = 10^−6^ and the full lines are for *h*_0_ = 10^−3^. (*b*) Convergence for quadrupole source. The dotted lines are for *h*_0_ = 10^−6^ and the full lines are for *h*_0_ = 10^−3^. In both cases, as expected, a larger *h*_0_ requires fewer degrees of freedom to achieve a given accuracy. (Online version in colour.)

5.1 $$h(x)=0.001x^{2}+h_{0},$$

for a small positive cut-off *h*_0_. If *h*_0_ = 0, the thin-plate equation (2.1) becomes singular at *x* = 0. For this reason, and to also avoid physically impractical cases, we consider examples of small but positive non-zero *h*_0_. We consider an incoming incident plane wave of angle 3*π*/4 and *k*_0_ = 20. Figure 9 shows the plate deformations for *h*_0_ = 10^−6^ and *h*_0_ = 10^−3^. For small *h*_0_, the plate vibrations become very large as the thickness decreases at *x* = 0. The oscillations become clustered near the thin portion of the plate (see the magnified section), and this effect is increased by making *h*_0_ smaller. This effect is removed when *h*_0_ = 10^−3^. Figure 10 shows the corresponding near fields. We see that near *x* = 0, the incident field is able to pass through the plate, causing little reflection for *h*_0_ = 10^−6^. Again this effect is removed for the larger *h*_0_ = 10^−3^. In figure 11, the far field is presented. There is a slight reduction in the scattered noise for smaller *h*_0_, with a less focused scattering direction. Finally, figure 12*a* plots the convergence of the physical variables of interest and demonstrates that we can easily gain several digits of relative accuracy, even for small *h*_0_.

### (b) Quadrupole sound source

The noise generated by the turbulence at the trailing edge of an aerofoil can make a significant contribution to the overall production of aeroacoustic noise, especially at high frequencies [57,58]. By Lighthill’s analogy, turbulent eddies are represented by a distribution of quadrupole sources in the same volume [59]. This motivated the study of a simplified model of the scattering by a plate with forcing given by a quadrupole at (*x*, *y*) = ( − 1, 0.001). The resulting near and far fields can be used to study aerofoil edge adaptions [30].

In this example, we take *α*_*H*_ ≡ 0 (i.e. zero porosity), and take the plate to be clamped at *x* = 1 but free at *x* = −1. The thickness is chosen to vary according to

5.2 $$h(x)=0.001{\left(x+1\right)}^{2}+h_{0},$$

for a small positive cut-off *h*_0_. We consider the case of *k*_0_ = 25 for *h*_0_ = 10^−6^ and *h*_0_ = 10^−3^. Convergence of the method is shown in figure 12*b*. We also checked our results near the vibrating tip by resorting to reciprocity. The value of the fields at (*x*, *y*) = ( − 1, 0.001) with a quadruple at (1, 1) is the same as the value at (*x*, *y*) = (1, 1) with the quadruple at ( − 1, 0.001).

**Figure 13.**
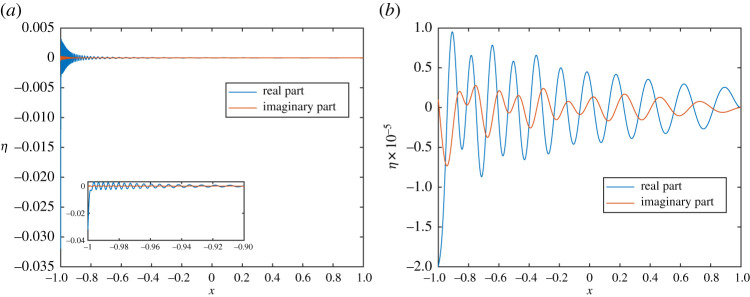
(*a*) Plate displacement, *k*_0_ = 25, for quadrupole sound source for acoustic black hole with *h*_0_ = 10^−6^. The magnified section shows the oscillatory waves near the plate tip. The (non-zero) imaginary component is small compared to the real component. (*b*) Results for *h*_0_ = 10^−3^. (Online version in colour.)

**Figure 14.**
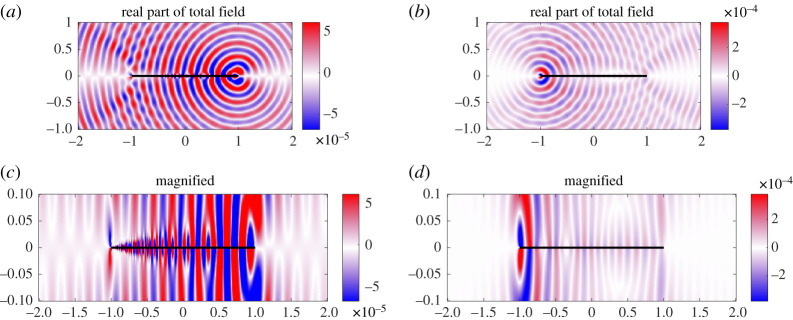
(*a*,*c*) Near field, *k*_0_ = 25, for quadrupole sound source for acoustic black hole with *h*_0_ = 10^−6^. (*b*,*d*) Results for *h*_0_ = 10^−3^. (Online version in colour.)

**Figure 15.**
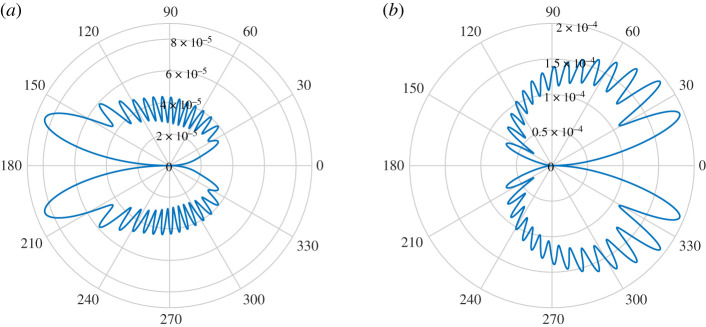
(*a*) Magnitude of the far-field directivity |*D*(*θ*)|, *k*_0_ = 25, for quadrupole sound source for acoustic black hole with *h*_0_ = 10^−6^. (*b*) Results for *h*_0_ = 10^−3^. (Online version in colour.)

Figures 13–15 show the plate deformations, near field and far field, respectively. The plate deformations behave qualitatively as before, with oscillations clustering near the thin part of the plate for smaller *h*_0_. The imaginary part of *η* for *h*_0_ = 10^−6^ is not zero, but it is small in comparison with the real part. This is because the real part of the incident quadrupole dominates near the source. We see a very interesting effect for the near field. The magnitude of the field is much smaller for *h*_0_ = 10^−6^, and in fact appears to be dominated locally around the right tip (1, 0) which is unusual. We can also see that there are evanescent pressure waves on the surface of the plate in figure 14 (magnified). The more flexible end of the plate absorbs (rather than scatters) the pressure fluctuations and propagates them down the plate to the less flexible endpoint. On reaching the *x* = 1 tip, the pressure fluctuations scatter resulting in a directivity pattern as if the main source was located near the *x* = 1 endpoint. By contrast, for *h*_0_ = 10^−3^, expected cardioid directivity around the point ( − 1, 0) is observed typical for such problems, and no evanescent pressure waves are visible. The corresponding pattern is observed in the far-field directivity, where we see that for *h*_0_ = 10^−6^, the scattered field is reflected back in the direction of the source and is much smaller than that of *h*_0_ = 10^−3^. The scattered field in the direction of the incident field is an interesting example of an acoustic black hole effect in a plate of varying elasticity. The authors are not aware of this effect being studied in such plates. The usual setting for this is an elastic wedge, where the cross-sectional thickness varies according to a power law [8]. One interesting observation is that the black hole effect relies on the power function going to nearly zero [8]. It is mitigated when the cross-sectional thickness decreases to 10^−3^ in figure 15*b*, where the majority of scattering obeys the usual reflection.

The above results indicate that there is potential to exploit the acoustic black hole effect in edge adaptations. For example, acoustic black holes can be used to direct the vibrations away from the aerofoil edges towards the middle of the aerofoil where vibration-absorbing mechanisms can be placed. The need for small *h*_0_ gives practical limitations for its use, but the truncated profiles with correctly placed damping layers can still be practical. There are some preliminary experimental studies for sound absorption in air [8]. However, currently, there is very little known about the use of acoustic black holes in aeroacoustics, and further theoretical and experimental investigations and validation are needed.

## 6. Extension to multiple plates

We can also use the numerical method in §3 to compute the scattered field from multiple plates. Suppose that we have plates *P*_[*i*]_ for *i* = 1, …, *S*, whose lengths are 2*d*_[*i*]_. We also suppose that the open set $R^{2}\setminus (\cup_{i=1}^{S}P_{\left[i\right]})$ is connected (in particular, we exclude the possibility that plates enclose a region, though this can be dealt with via suitable modifications). We use sub/superscripts [*i*] to denote quantities associated with the plate *P*_[*i*]_. Each plate *P*_[*i*]_ induces a corresponding scattered field given by

6.1 $$\phi_{\left[i\right]}(x,y)=\sum_{m=1}^{\infty }a_{m}^{\left[i\right]}{\text{se}}_{m}(Q_{\left[i\right]};\tau_{\left[i\right]}){\text{Hse}}_{m}(Q_{\left[i\right]};\nu_{\left[i\right]}),$$

where (*ν*_[*i*]_, *τ*_[*i*]_) = (*ν*_[*i*]_(*x*, *y*), *τ*_[*i*]_(*x*, *y*)) are elliptic coordinates centred around *P*_[*i*]_, and $Q_{\left[i\right]}=d_{\left[i\right]}^{2}k_{0}^{2}/4$. The total scattered field is given by the sum of these contributions $\phi =\sum_{i=1}^{S}\phi_{\left[i\right]}$ and along each plate we apply poro-elastic boundary conditions as before. Clearly *ϕ* satisfies the Helmholtz equation and Sommerfeld radiation condition.

Numerically, we solve this problem in the same way, where we take *M*_[*i*]_ Mathieu functions for the expansion along the *i*th plate and we supplement the expansion of *ϕ*_[*i*]_ with an expansion of *η*_[*i*]_ in terms of *N*_[*i*]_ Chebyshev polynomials of the first kind along the plate *P*_[*i*]_. The relation (2.1) becomes

6.2 $$\begin{array}{rl} & \sum_{j=0}^{N_{\left[i\right]}-1}{\tilde{b}}_{j}^{\left[i\right]}\sum_{l=0}^{4}\frac{B_{l}^{\left[i\right]}(x_{\left[i\right]})}{d_{\left[i\right]}^{l}}T_{j}^{\left(l\right)}\left(\frac{x_{\left[i\right]}}{d_{\left[i\right]}}\right)\\ & \;+2\rho_{f}c_{0}^{2}\left(1+\frac{4\alpha_{H}^{\left[i\right]}(x_{\left[i\right]})}{\pi }\right)\sum_{m=1}^{M_{\left[i\right]}}{\tilde{a}}_{m}^{\left[i\right]}{\text{se}}_{m}\left(Q_{\left[i\right]};\cos^{-1}\left(\frac{x_{\left[i\right]}}{d_{\left[i\right]}}\right)\right){\text{Hse}}_{m}(Q_{\left[i\right]};0)=0, \end{array}$$

where *x*_[*i*]_ denotes a unit speed parametrization of the plate *P*_[*i*]_ for *x*_[*i*]_ ∈ [ − *d*_[*i*]_, *d*_[*i*]_]. We collocate this relation for (*x*, *y*) points corresponding to *N*_[*i*]_ − 4 Chebyshev points along *P*_[*i*]_ (so that *x*_[*i*]_/*d*_[*i*]_ correspond to standard Chebyshev points). Again, we supplement this system with four relations enforcing the boundary conditions at *x*_[*i*]_ = ±*d*_[*i*]_. The kinematic relation (2.2) becomes

6.3 $$\begin{array}{rl} & \sum_{m=1}^{M_{i}}{\tilde{a}}_{m}^{\left[i\right]}{\text{se}}_{m}\left(Q_{\left[i\right]};\cos^{-1}\left(\frac{x_{\left[i\right]}}{d_{\left[i\right]}}\right)\right)\left[1-\frac{4\alpha_{H}^{\left[i\right]}(x_{\left[i\right]}){\text{Hse}}_{m}(Q_{\left[i\right]},0)}{\pi R_{\left[i\right]}(x_{\left[i\right]})}\sqrt{d_{\left[i\right]}^{2}-x_{\left[i\right]}^{2}}\right]\\ & \;-k_{0}^{2}(1-\alpha_{H}^{\left[i\right]}(x_{\left[i\right]}))\sqrt{d_{\left[i\right]}^{2}-x_{\left[i\right]}^{2}}\sum_{j=0}^{N_{\left[i\right]}-1}{\tilde{b}}_{j}^{\left[i\right]}T_{j}\left(\frac{x_{\left[i\right]}}{d_{\left[i\right]}}\right)\\ & \;=-\sqrt{d_{\left[i\right]}^{2}-x_{\left[i\right]}^{2}}\cdot \frac{\partial }{\partial y}\left[\phi_{\text{I}}+\sum_{j\neq i}\sum_{m=1}^{M_{\left[j\right]}}a_{m}^{\left[j\right]}{\text{se}}_{m}(Q_{\left[j\right]};\tau_{\left[j\right]}){\text{Hse}}_{m}(Q_{\left[j\right]};\nu_{\left[j\right]})\right](x,y), \end{array}$$

and we collocate at *M*_[*i*]_ Chebyshev points along *P*_[*i*]_. The above collocated relations generate a square $\left(\sum_{i=1}^{S}M_{\left[i\right]}+N_{\left[i\right]})\times (\sum_{i=1}^{S}M_{\left[i\right]}+N_{\left[i\right]}\right)$ linear system which we solve for the approximate coefficients in the expansion. For large *S*, an iterative method of solution rather than solving the full coupled system directly may be more numerically efficient (as was found to be the case for a Wiener–Hopf method tackling rigid non-porous plates [41]), but we found a simple direct approach to be effective for moderate values of *S*. Future work will also look at fast multipole methods and hierarchical solvers for multiple plates and evaluation of the solutions.

As a simple example, we consider the case of two plates where *P*_[1]_ is elastic and clamped with endpoints ( ± 1, *L*) and *P*_[2]_ is rigid with endpoints ( ± 1, − *L*). For *P*_[1]_, we set

6.4 $$B_{4}(x)=B,\;B_{3}(x)=B_{2}(x)=B_{1}=0,\;B_{0}(x)=-\omega^{2},$$

where a constant stiffness has been chosen so that we were able to validate the results with the methods of [9]. We consider a plane wave incident field of angle *π*/4 and *k*_0_ = 10.

**Figure 16.**
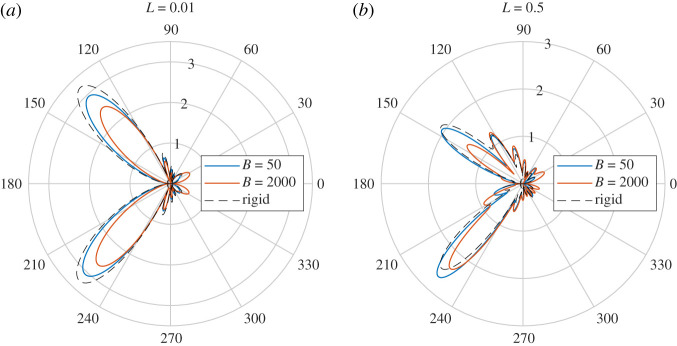
The far fields for *L* = 0.01 (*a*) and *L* = 0.5 (*b*). We have also shown the rigid case for comparison. (Online version in colour.)

**Figure 17.**
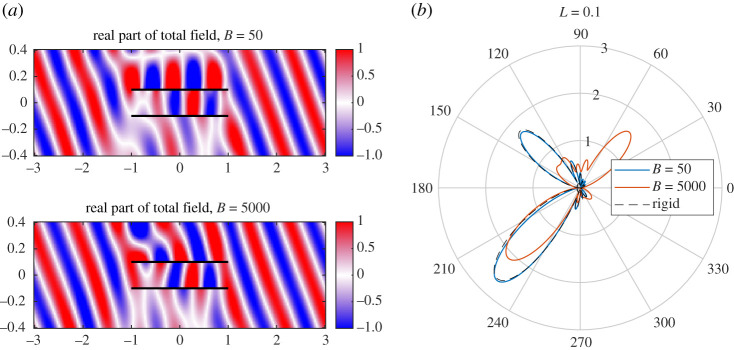
(*a*) The near fields for *L* = 0.1 and corresponding duct modes. (*b*) The far fields for *L* = 0.1. (Online version in colour.)

Figure 16 shows the far fields for *L* = 0.01 and *L* = 0.5. In the acoustic compact case $L\ll k_{0}^{-1}$, the scattered field behaves as if it is incident on a single plate. This gives a symmetric scattered field, which does not vary monotonically with *B* (as expected due to effects such as resonances). For larger spacings, each edge (four in total) scatters an acoustic field which interacts in the far field to create an oscillatory directivity pattern. If the elastic plate is suitably flexible to be excited by the incident wave and absorb energy, its scattering will be distinctly different to a rigid plate, and hence alter the overall far field directivity. The primary effects are noticeable in the Fresnel lobes. Figure 17 shows the near field and far field for $L=0.1=O(k_{0}^{-1})$. In this case, the plates support a specific ‘duct’ mode between them. The scattering of these modes by the edges contributes to the far-field noise. Altering the elasticity of the upper plate alters the fundamental structure of the duct and what modes can exist there. This too impacts the scattering in addition to direct scattering by each of the four edges.

## 7. Conclusion

This article developed a boundary spectral method, based on collocation of local Mathieu function expansions, for Helmholtz scattering off multiple variable poro-elastic plates. Such boundary conditions are challenging for current methods, and we compared our approach to an elastic boundary element method in §3c, where it was found to be considerably faster and more accurate. Moreover, previous use of Mathieu functions has been limited to constant physical parameters and small degrees of expansions. By contrast, we were able to compute expansions in thousands (and even tens of thousands) of Mathieu functions by making use of the Bessel function expansion of Mathieu–Hankel functions and their asymptotics. This allows quick and robust testing of physical parameters and variations, which may have use in other scattering problems beyond those considered here.

We found that the method coped well with a broad range of frequencies (typically needing more terms for larger *k*_0_, as expected) and smoothly varying porosity/elasticity (with more collocation points and Mathieu functions needed to capture the case of more oscillatory parameters). Our solution representation also directly provides a sine series approximation of the far-field directivity and, unlike standard boundary methods, can easily be evaluated near or on the scatterers. This means that the acoustic near field can be computed efficiently and in a stable manner. These advantages assert that the present method is particularly good for a simple model of turbulence using Lighthill’s analogy.

Examples of diffraction by elastic plates of varying stiffness were presented. We found that a plate with varying stiffness can exhibit an acoustic black hole type behaviour. This has a drastic effect on the near and far fields, both in the scattering and the aeroacoustics setting. Further work is needed to understand how this might be employed as a leading or a trailing edge adaptation to an aerofoil. There is also a potential to use this acoustic black hole effect to move the vibrations away from the trailing edge and into the centre of an aerofoil where they can be baffled.

Finally, we demonstrated that the numerical method can be used on multiple, arbitrary positioned plates. Future work will also look at fast multipole methods and hierarchical solvers for multiple plates and evaluation of the solutions. The method also offers considerable flexibility in the choice of forcing term. In this article, we only considered plane waves and quadrupoles. The new method can easily be extended to boundary conditions different to those in §2 (such as linking different parts of the scatterer or integral constraints) and can be generalized to include boundary conditions on ellipses. While the method is currently restricted to finite plates in two dimensions, it may also be possible to consider similar approaches to other problems (e.g. three dimensions) through separation of variables and different special functions accompanied by spectral methods.

## Supplementary Material

A Mathieu function boundary spectral method for scattering by multiple variable poro-elastic plates, with applications to metamaterials and acoustics: Supplementary material

## Footnotes

1 A comparison of the new extensions of the unified transform [39], iterative Wiener–Hopf method [40,41] and Mathieu functions solution for a porous plate has recently been performed in [42].

2 There is an additional factor of (1 − *α*_*H*_) compared to [30] due to our direct use of the boundary conditions in [46]. This was absorbed into the definition of *m* in [30].

## Data accessibility

This work does not contain any experimental data, and all of the results can easily be generated from the equations provided in the article. Numerical code is available at https://github.com/MColbrook/MathieuFunctionCollocation.
