# Supplementary material for: A Mathieu function boundary spectral method for scattering by multiple variable poro-elastic plates, with applications to metamaterials and acoustics
Source: Proc Math Phys Eng Sci. 2020 Sep 23;476(2241):20200184. doi: 10.1098/rspa.2020.0184 (PMC7544365; doi:10.1098/rspa.2020.0184)
Supplement: A Mathieu function boundary spectral method for scattering by multiple variable poro-elastic plates, with applications to metamaterials and acoustics: Supplementary material [file rspa20200184supp1.pdf]

PROCEEDINGS A

rspa.royalsocietypublishing.org

Research

Article submitted to journal

Keywords:
boundary spectral methods, Mathieu functions, acoustic scattering, poro-elastic boundary conditions, metamaterials

Author for correspondence:
Matthew Colbrook
e-mail: m.colbrook@damtp.cam.ac.uk

A Mathieu function boundary spectral method for scattering by multiple variable poro-elastic plates, with applications to metamaterials and acoustics: Supplementary material

Matthew J. Colbrook*, Anastasia V. Kisil†

*Department of Applied Mathematics and Theoretical Physics, University of Cambridge, Wilberforce Road, CB3 0WA, UK
† Department of Mathematics, The University of Manchester, Manchester, M13 9PL, UK

Method for rigid porous plate

For completeness, as a reference for the reader, and to demonstrate the ease of adopting other types of boundary conditions, we discuss the case of rigid porous plates.

The porous plate impedance boundary condition is given by

$$\frac{\partial \phi}{\partial y} + \frac{\partial \phi_{\mathrm{I}}}{\partial y} = \mu(x)\left(\phi(x,0+) - \phi(x,0-)\right) = \mu(x)[\phi](x), \tag{0.1}$$

where $\mu = \alpha_H K_R/(\pi R^2)$ is the porosity parameter for a porous plate with evenly-spaced circular apertures of radius R, Rayleigh conductivity of $K_R = 2R$, and fractional open area $\alpha_H = N\pi R^2$ (where N is the number of apertures per unit area) [4]. As in the main article, $\phi(x,0+)$ and $\phi(x,0-)$ denote the values of the field just above and just below the plate respectively and the jump in ϕ across the plate is denoted by $[\phi](x)$. We also allow the porosity parameter, $\mu(x)$, to vary across the plate. The boundary condition (0.1) replaces the thin-plate equation and the kinematic condition for the elastic plates considered in the main text.

The solution method is exactly the same, but now we use collocation to solve the boundary condition (0.1). We truncate the Mathieu function expansion to M terms and collocate at points x to obtain the relation

THE ROYAL SOCIETY PUBLISHING

© The Author(s) Published by the Royal Society. All rights reserved.

$$\sum_{m=1}^{M} \tilde{a}_m \mathrm{se}_m \left(\cos^{-1}\left(\frac{x}{d}\right)\right) \left[1 - 2\mathrm{H}\mathrm{se}_m(0)\mu(x)\sqrt{d^2 - x^2}\right] = -\sqrt{d^2 - x^2} \cdot \frac{\partial \phi_{\mathrm{I}}}{\partial y}(x).$$

For collocation points, we choose

$$\left\{ d\cos\left(\frac{2j-1}{2M}\pi\right) : j = 1, \ldots, M \right\},$$

which correspond to (rescaled) Chebyshev points in Cartesian coordinates and equally spaced points in elliptic coordinates [1, 7]. This gives rise to an $M \times M$ linear system. As in the main text, we rescale to ensure that each row of the resulting matrix has a constant l^1 vector norm. The method can also be extended to multiple plates with a mixture of different boundary conditions.

Details for separation of variables for a single plate

We introduce elliptic coordinates via $x = d\cosh(\nu)\cos(\tau), y = d\sinh(\nu)\sin(\tau)$, where, with an abuse of notation, we write functions of (x, y) also as functions of (ν, τ). The appropriate domain then becomes $\nu \geq 0$ and $\tau \in [0, \pi]$. The appropriate domain then becomes $\nu \geq 0$ and $\tau \in [0, \pi]$, and the PDE system (now with a homogeneous Dirichlet boundary condition along $\{(x, y) : y = 0, |x| > d\}$) becomes

$$\begin{cases} \frac{\partial^2 \phi}{\partial \tau^2} + \frac{\partial^2 \phi}{\partial \nu^2} + \frac{\cosh(2\nu) - \cos(2\tau)}{2} d^2 k_0^2 \phi = 0, \\ \phi|_{\tau=0} = \phi|_{\tau=\pi} \equiv 0, \\ \lim_{\nu \to \infty} \nu^{1/2} \left(\frac{\partial}{\partial \nu} - \mathrm{i}dk_0\right) \phi(\nu, \tau) = 0. \end{cases}$$

To simplify the formulae, we let $Q = d^2 k_0^2 / 4$. Separation of variables for solutions of the form $V(\nu)W(\tau)$ leads to the regular Sturm–Liouville eigenvalue problem

$$W''(\tau) + (\lambda - 2Q\cos(2\tau))\, W(\tau) = 0, W(0) = W(\pi) = 0.$$

The solutions of this are sine-elliptic functions, denoted by se_m with eigenvalue λ_m, which we expand in a sine series as

$$\mathrm{se}_m(Q; \tau) = \mathrm{se}_m(\tau) = \sum_{l=1}^{\infty} B_l^{(m)} \sin(l\tau). \tag{0.2}$$

This Fourier series converges absolutely and uniformly on all compact sets of the complex plane [6]. The eigenfunctions are real and orthogonal, and we choose the normalisation

$$\int_0^{\pi} \mathrm{se}_m(\tau)\mathrm{se}_n(\tau) d\tau = \frac{\pi}{2}\delta_{mn}.$$

We find the coefficients $B_l^{(m)}$ via a simple Galerkin method. Namely, we split the eigenfunctions further by symmetry or antisymmetry about $\tau = \pi/2$ and write

$$\mathrm{se}_{2m}(\tau) = \sum_{l=1}^{\infty} B_{2l}^{(2m)} \sin(2l\tau),$$

$$\mathrm{se}_{2m+1}(\tau) = \sum_{l=0}^{\infty} B_{2l+1}^{(2m+1)} \sin((2l+1)\tau).$$

For the even order solutions, the eigenvalue problem becomes the tridiagonal system

$$\begin{pmatrix} 2^2 - \lambda_{2m} & Q & & \\ Q & 4^2 - \lambda_{2m} & Q & \\ & Q & 6^2 - \lambda_{2m} & Q \\ & & \ddots & \ddots & \ddots \end{pmatrix} \begin{pmatrix} B_2^{(2m)} \\ B_4^{(2m)} \\ B_6^{(2m)} \\ \vdots \end{pmatrix} = 0.$$

A similar system holds for the odd order solutions:

$$\begin{pmatrix} 1^2-\lambda_{2m+1}-Q & Q & & & \\ Q & 3^2-\lambda_{2m+1} & Q & & \\ & Q & 5^2-\lambda_{2m+1} & Q & \\ & & \ddots & \ddots & \ddots \end{pmatrix} \begin{pmatrix} B_1^{(2m+1)} \\ B_3^{(2m+1)} \\ B_5^{(2m+1)} \\ \vdots \end{pmatrix} = 0.$$

These are solved using square $n \times n$ truncations of the infinite matrix (also known as the finite section method or Galerkin method). Since the spectrum of the associated (self-adjoint) linear operator is discrete, we do not have to worry about issues such as spectral pollution [3]. The convergence to the eigenvalues and eigenfunctions depends on the parameter Q, in general being slower for larger Q. However, the convergence is exponential, yielding machine precision for small truncation parameter n, even for very large Q [2].

The corresponding $V(\nu)$ with the appropriate radiation condition at infinity are given by the Mathieu–Hankel functions

$$\mathrm{Hse}_m(Q;\nu) = \mathrm{Hse}_m(\nu) = \mathrm{Jse}_m(\nu) + \mathrm{i}\mathrm{Yse}_m(\nu),$$

which can be expanded in a series using Bessel functions as in the main text.

References

1 J. P. Boyd, *Chebyshev and Fourier spectral methods*, Courier Corporation, 2001.

2 M. J. Colbrook and M. J. Priddin, *Fast and spectrally accurate numerical methods for perforated screens (with applications to robin boundary conditions)*, Preprint.

3 M. J. Colbrook, B. Roman, and A. C. Hansen, *How to compute spectra with error control*, Physical Review Letters **122** (2019), no. 25, 250201.

4 M. S. Howe, *Acoustics of fluid-structure interactions*, Cambridge Monographs on Mechanics, Cambridge University Press, 1998.

5 F. W. J. Olver, D. W. Lozier, R. F. Boisvert, and C. W. Clark, *NIST handbook of mathematical functions*, Cambridge university press, 2010.

6 L. N. Trefethen, *Spectral methods in MATLAB*, vol. 10, Siam, 2000.
